# Gender Norms and Gender Equality in Full-Time Employment and Health: A 97-Country Analysis of the World Values Survey

**DOI:** 10.3389/fpsyg.2022.689815

**Published:** 2022-05-31

**Authors:** Beniamino Cislaghi, Amiya Bhatia, Emma Sofia Thonander Hallgren, Nour Horanieh, Ann M. Weber, Gary L. Darmstadt

**Affiliations:** ^1^Department of Global Health and Development, London School of Hygiene and Tropical Medicine, London, United Kingdom; ^2^Center for Population Health Sciences, Stanford University School of Medicine, Palo Alto, CA, United States; ^3^School of Community Health Sciences, University of Nevada, Reno, Reno, NV, United States; ^4^Department of Pediartrics, Stanford University School of Medicine, Stanford, CA, United States

**Keywords:** gender equality, gender norms, women empowerment, full-time employment, self-reported health (SRH)

## Abstract

**Background:**

Almost nowhere in the world do women participate as much as men in the labor force. Despite differences in countries’ economic, social and cultural contexts, gender norms—unwritten rules of acceptable actions for men and women—have been found to affect women’s labor participation across contexts. Gender norms include those regulating who takes care of children, who is expected to earn more, and in which sectors men and women should work. Importantly, norms affect access to labor markets at times of scarcity: when there’s only work for one, gender norms can dictate whether a woman or man gets the job. Advocates of equal labor force participation point to evidence that employment can contribute to people’s health and well-being; yet the evidence is mixed and contradictory, and mostly comes from high-income countries. In restrictive normative contexts in which women are assigned the role of family caretaker, full time employment (FTE) might be particularly burdensome. At the same time, the literature lacks a cross-country analysis of how gender norms affect women’s FTE and their health when employed full time, despite qualitative research providing clear evidence of the influence of gender norms on labor participation.

**Aims:**

In this paper we examine: (1) how gender norms affect women’s access to FTE across 97 countries; (2) associations between FTE and women’s self-reported health self-rated (SRH) across different normative contexts (i.e., countries where it is common vs. uncommon for women to stay home); and (3) how women’s FTE and gender norms changed over time in four countries.

**Data:**

We used time-series data from the World Values Survey and European Values Survey conducted in over 100 countries between 1981 and 2014. Both surveys attempt to capture norms, beliefs and values in addition to sociodemographic information among a nationally representative adult population in each country. The sample for the cross-sectional analyses (aims 1 and 2) included 97 countries and 131,132 respondents. The sample for aim 3 included data from Argentina, Egypt, Finland and Japan.

**Variables:**

Our outcome of interest was pro-equality norms in the context of access to the labor market for women. Respondents were asked “if jobs are scarce, men should have more right to a job than women do?”. Response options included no, neither or yes. We created a binary variable to represent pro-equality norms. We included employment status and SRH as exposures of interest.

**Analysis:**

We used individual-level data to generate on-average and sex-stratified estimates of the outcome and exposures for each country, at each time point. We estimated the percentage of all respondents, of women, and of men who held pro-equality norms (believe that men should not have more right to a job than women), the percentage who were employed full time, and the average level of SRH. To measure gender inequality in FTE, we also estimated the absolute difference in FTE between women and men for each country at each time point. First, we conducted descriptive, cross-sectional ecological analyses using one survey per country from wave 5 or 6 (whichever was most recent) to examine associations between pro-equality norms and employment status as a proxy for associations between norms and the context of employment in each country. We also examined associations between pro-equality norms and SRH. We then specified adjusted logistic regression models with controls for age, sex and education to examine associations between pro-equality norms and employment status. To examine if the relationship between FTE and SRH varied by normative context, we grouped countries in quartiles of pro-equality norms. Finally, we conducted descriptive ecological analyses of the relationship between pro-equality norms and employment status over time in four countries.

**Results:**

Objective 1: Gender norms intersect with socio-cultural contexts in determining women’s FTE. While in some countries gender norms aligned positively with women’s access to employment (i.e., more equal norms matched more equality in FTE), in Eastern Europe and South America we observed a mismatch. In Eastern Europe we found strong norms against equal access but small sex differences in FTE. In South America, we observed a stark difference in FTE favoring men, despite positive gender norms promoting women’s paid employment. Objective 2: We found the association between SRH and FTE to vary across normative contexts. For instance, while in Scandinavian countries it was protective to be a woman in FTE and harmful not to work full-time, we found the opposite effect in Middle Eastern countries. Objective 3: We found a general tendency to move toward greater equality in norms and FTE over time everywhere in the world. However, political and economic events can generate variations over time and setbacks in progress toward equality.We specifically looked at 4 countries: Argentina, Egypt, Finland and Japan and assessed the effects of economic, political and national legislative changes on FTE over time.

**Implications:**

This paper contributes to the conversation on tensions between universal justice and contextual factors affecting one’s health. To achieve purposeful and global universal health and justice, policy makers and global health practitioners must design effective, context-relevant interventions that are deeply and transparently informed by the values they embody. As we strive to achieve global gender equality, its meanings and purposes will vary across contexts in ways that demand people-led conversations and interventions.

## Introduction

Gender equality is both a global aim in itself as well as a means to achieve other Sustainable Development Goals (Stevens, 2015; Esquivel and Sweetman, 2016; Fredman et al., 2016). Achieving greater gender equality can yield positive results across several areas of life, including health, rights, and wellbeing (Taukobong et al., 2016; Solé-Auró et al., 2018; Roxo et al., 2020; Sudkämper et al., 2020), but several challenges exist to achieving gender equality. These challenges include a plethora of deeply entrenched practices and beliefs in the form of discriminatory government laws (Doepke et al., 2012), institutional policies (Jayachandran, 2020), unequal access to resources (Bishu and Alkadry, 2017), unfair marriage and divorce practices (Iversen et al., 2005; Schoon et al., 2005; Genadek et al., 2007), or discriminatory service availability and access (Roy, 2019). Although gender inequalities affect the lives of people of all genders, we focus in our analysis on how it affected women and men, due to restricted availability of only binary data.

To measure gender equality at country level, researchers and policy makers often use, in combination with other indicators, women’s and men’s equal participation in full-time employment (FTE) (Bardhan and Klasen, 1999). Reasons for the choice of equal employment are found both in cross-country data availability and in the fact that FTE can affect people’s health and well-being in profound ways (Roxo et al., 2020). For instance, compared to people who are not in FTE, full-time workers may experience an increased feeling of importance, a larger social network and greater economic independence (Clark et al., 2008; Bambra, 2010; Rosenthal et al., 2012). Gender inequality in FTE has been linked to myriad institutional, material and social factors. A few examples include: unequal access to education—despite a reducing gap and an reversed gap advantaging women in certain countries (Klasen and Lamanna, 2009); national labor policies such as lack of paternal leave which forces women to take extended time off work (Ciccia and Verloo, 2012; Castro-García and Pazos-Moran, 2016); religious practices that discourage women’s work outside of the household (Tlaiss, 2014); lack of safe and accessible transportation for women (Alkadry and Tower, 2006), and gender pay gaps that make it preferable for heterosexual couples to have men keep their jobs when someone is needed at home (Wolf et al., 2016).

Yet, even when some of these structural and material factors are addressed (that is, for instance, the education gap is reduced, paternal leave is introduced or the pay gap is narrowed), women’s FTE can still lag behind men’s. A possible explanation for the lack of a swift change after reduction of structural barriers is that material and institutional factors are but one part of the problem; social factors present less tangible yet very real hurdles to women’s full-time participation in the labor force. Among these social factors, gender norms, the unwritten rules of (un)acceptable actions for men and women (Cislaghi and Heise, 2019), are considered one of the most important, due to their ubiquitousness and power. Gender norms give rise to behavioral expectations for people of a given gender. These expectations can limit women’s access to employment; take, for instance, the norms that women should take care of children and family members (Jayachandran, 2020), that men should earn more than women to be real men (Thebaud, 2010), or that women and men should engage in certain professional occupations (for instance, nursing for women and managing hedge funds for men), but not others (being a surgeon for women and being a babysitter for men) (Couch and Sigler, 2001; Yasukawa and Nomura, 2014). Gender norms mostly exert their influence through anticipation of negative sanctions (scolding, gossiping, ostracization, violence, for instance) for non-compliers. The anticipation of sanctions reduces the chances that someone might deviate from these norms, limiting visibility of alternative behavioral models. Analyzing data from Nigeria on female access to labor and their experience of violence, Weber et al. (2019) found that women who contravened gender norms that made it unacceptable for women to work outside of the household were at significantly higher risk of experiencing intimate partner violence compared to those women who did not contravene the gender norm (Weber et al., 2019).

Using data from the World Value Survey (WVS) and the European Value Surveys (EVS), in this paper we look at men’s and women’s participation in the labor market as a case study to understand links between gender norms, women’s participation in FTE, and health. Specifically, this paper examines gender norms, FTE for men and women, and self-reported health (SRH) in 97 countries. We explore the extent to which access to FTE affects SRH across different normative contexts (i.e., countries where it is common vs. uncommon for women to stay home) and, for a sample of case-study countries, we examine how gender norms, FTE and SRH have changed over time and potential ecological factors shaping these trends.

## Materials and Methods

### Data Sources

Data for this study come from the WVS and EVS (Inglehart et al., 2014a,b), both of which capture norms, beliefs and values in addition to sociodemographic information among nationally representative adult populations in each country. The data are captured in waves (of which the sixth was the most recently available)ranging over a span of approximately ten years. We used publicly available data from the WVS website: the integrated dataset combined four waves of EVS in 47 countries and six waves of WVS surveys all conducted between 1981 and 2014 in 100 countries (World Values Survey, 2019).

In our analysis, we included 97 countries for which data were collected in wave 5 or 6 (using data from the most recent wave), with at least 3 waves of data (to assess change over time) and with information on our three variables of interest: (1) gender norms about job scarcity, (2) employment status, and (3) SRH. We restricted our sample to respondents between 20 and 70 years of age, excluding those who were either too young or too old to be in FTE to account for the range of ages at which people enter and leave the labor market across the countries in our sample.

### Variables

Our first variable of interest was gender norms about job scarcity. Respondents were asked “*if jobs are scarce, men should have more right to a job than women do?*” Response options included no, neither or yes. We created a binary variable to represent pro-equality norms (PEN). The denominator included all respondents who answered the question and the numerator was all respondents who answered “no” who were coded as having pro-equality norms. Our second variable of interest, full-time employment (FTE), was a binary variable that included all respondents who reported they were working full time. Respondents who were working part-time, were self-employed, retired, students, house-wives or unemployed or answered ‘other’ were coded as not in FTE. Finally, we included SRH as a categorical variable (1 = Very poor, 2 = Poor, 3 = Fair, 4 = Good, 5 = Very good).

### Analyses

We used individual-level data to generate on-average and sex-stratified estimates of each variable for each country. We calculated the percentage of male and female respondents who held PEN, the percentage in FTE, and the average level of SRH. To measure gender inequality in FTE, we calculated the absolute percentage-point difference between men and women for each country; Negative values indicated that fewer women were employed full time. We also determined average SRH for men and women, and gender inequality in SRH, by FTE status.

Descriptive cross-sectional ecological analyses were conducted using one survey per country from wave 5 or 6 (whichever was most recent) to describe PEN, FTE, SRH and gender inequalities in employment and health in each country. To examine if the relationship between FTE and SRH varied by normative context, we grouped countries into quartiles of PEN and descriptively examined employment status and SRH within each quartile. For each country, we also specified logistic regression models to examine adjusted associations between PEN (dependent variable) and FTE (independent variable). Covariates included: age (in 10-year age groups) and education level (lower, middle, upper) as ordinal variables, and sex (male, female) as a binary variable.

Finally, we examined changes over time in PEN, FTE, and SRH for men and women in four countries: Argentina, Egypt, Finland and Japan. We used the results of the cross sectional analysis to select these countries for more detailed analysis of the historical and policy context. Countries were selected among those for which we had at least three waves of data to include examples from different regions, and with different levels of gender inequality in FTE and PEN. For the case studies, we conducted a search of the relevant literature, extracting historical information that was synthesized in the relevant section. All analyses were conducted using Stata 16. Analyses were unweighted as weights were unavailable for many countries in the sample.

## Results

### Sample Characteristics

The sample for the cross-sectional analyses included 97 countries and data from 131,132 respondents. The time trend analysis included 4 countries. Table 1 presents average PEN, FTE and SRH for the most recent survey in each country. Sample sizes ranged from 653 to 3158 (median = 1,232) for PEN; 660 to 3,167 (median = 1,239) for FTE; and 659 to 3180 (median = 1,260) for SRH. Supplementary Table 1 includes sample characteristics (age, education, and employment status).

**TABLE 1:** Pro-equality norms, full-time employment and self-reported health in 97 countries (men and women combined).

Region	Country	Survey Year	Sample size	Pro-equality norms (PEN)			Full-time employment (FTE)			Self-reported health (SRH)		
				%	SEM	*n*	%	SEM	*n*	Mean	SEM	*n*
Africa	Algeria	2013	1,075	20.0	1.24	1,040	21.9	1.26	1,075	3.8	0.03	1,058
	Burkina	2007	1,270	35.1	1.36	1,226	11.0	0.90	1,211	3.9	0.02	1,260
	Egypt	2013	1,384	7.9	0.72	1,384	16.3	0.99	1,384	3.6	0.03	1,384
	Ethiopia	2007	1,349	84.6	0.99	1,325	24.7	1.17	1,349	3.8	0.02	1,346
	Ghana	2012	1,340	45.2	1.36	1,340	23.7	1.16	1,340	4.4	0.02	1,340
	Libya	2014	2,013	16.6	0.83	1,992	24.6	0.97	1,987	4.3	0.02	2,002
	Mali	2007	1,258	22.5	1.19	1,232	10.6	0.88	1,227	3.8	0.03	1,237
	Morocco	2011	1,155	31.9	1.39	1,124	28.8	1.33	1,155	4.0	0.03	1,155
	Nigeria	2011	1,596	22.7	1.05	1,596	13.7	0.86	1,596	4.5	0.02	1,596
	Rwanda	2012	1,471	35.4	1.25	1,471	18.3	1.01	1,471	4.1	0.02	1,471
	South Africa	2013	3,182	48.1	0.89	3,158	34.5	0.85	3,167	4.2	0.01	3,180
	Tunisia	2013	1,053	17.7	1.19	1,028	24.1	1.32	1,053	3.9	0.03	1,052
	Zambia	2007	1,228	51.8	1.45	1,195	22.6	1.19	1,228	3.9	0.03	1,188
	Zimbabwe	2012	1,366	56.4	1.34	1,366	24.7	1.17	1,366	4.3	0.02	1,366
Americas	Argentina	2006	851	64.9	1.65	833	36.3	1.65	851	4.1	0.03	850
	Brazil	2014	1,317	74.4	1.21	1,309	34.9	1.31	1,317	3.9	0.02	1,316
	Canada	2006	1,754	82.0	0.92	1,743	43.5	1.19	1,749	4.2	0.02	1,750
	Chile	2011	886	58.8	1.67	874	49.5	1.68	886	3.9	0.02	885
	Colombia	2012	1,337	65.4	1.30	1,334	28.7	1.24	1,337	4.0	0.02	1,336
	Ecuador	2013	1,058	55.0	1.53	1,057	32.1	1.44	1,058	4.0	0.02	1,058
	Guatemala	2004	917	71.2	1.50	911	52.3	1.66	904	3.8	0.03	917
	Mexico	2012	1,761	71.3	1.08	1,757	26.4	1.05	1,757	4.0	0.02	1,760
	Peru	2012	1,046	65.6	1.49	1,016	30.5	1.42	1,046	3.6	0.02	1,045
	Trinidad	2011	854	62.8	1.67	842	43.6	1.70	853	4.2	0.03	854
	United States	2011	1,929	71.3	1.04	1,910	48.4	1.15	1,890	4.1	0.02	1,915
	Uruguay	2011	826	69.1	1.62	815	47.3	1.74	822	4.1	0.03	825
Asia	Armenia	2011	900	35.7	1.60	899	27.7	1.49	900	3.4	0.03	898
	Azerbaijan	2011	903	9.3	0.97	903	46.7	1.66	903	3.7	0.03	903
	Bahrain	2014	1,150	18.8	1.16	1,135	19.4	1.17	1,150	3.9	0.02	1,142
	China	2012	2,137	40.4	1.10	1,999	53.2	1.08	2,137	3.8	0.02	2,126
	Cyprus	2011	882	53.7	1.68	882	49.0	1.68	882	4.0	0.03	881
	Georgia	2014	1,030	44.6	1.55	1,029	20.4	1.26	1,029	3.4	0.03	1,030
	Hong Kong	2013	861	41.6	1.68	859	45.2	1.70	859	3.7	0.03	858
	India	2014	1,497	17.9	0.99	1,491	14.0	0.93	1,394	4.0	0.02	1,491
	Indonesia	2006	1,834	35.6	1.13	1,801	33.5	1.10	1,833	3.9	0.02	1,823
	Iran	2007	2,314	15.9	0.76	2,300	20.2	0.84	2,303	3.8	0.02	2,278
	Iraq	2012	1,095	17.6	1.15	1,090	20.4	1.22	1,095	3.7	0.02	1,090
	Japan	2010	2,047	16.6	0.85	1,928	48.2	1.13	1,967	3.6	0.02	2,008
	Jordan	2014	1,048	13.0	1.04	1,048	20.2	1.24	1,048	4.1	0.03	1,048
	Kazakhstan	2011	1,348	28.2	1.23	1,348	50.2	1.36	1,348	3.7	0.02	1,348
	Kuwait	2014	1,201	19.7	1.16	1,181	50.7	1.45	1,188	4.3	0.02	1,191
	Kyrgyzstan	2011	1,367	24.2	1.16	1,366	26.4	1.19	1,366	3.9	0.02	1,365
	Lebanon	2013	1,092	36.2	1.46	1,083	33.2	1.43	1,092	4.0	0.02	1,089
	Malaysia	2012	1,194	17.9	1.11	1,194	50.2	1.45	1,194	4.2	0.02	1,194
	Pakistan	2012	1,110	21.3	1.23	1,107	20.0	1.20	1,110	4.1	0.03	1,105
	Palestine	2013	902	22.1	1.39	893	21.6	1.37	902	4.0	0.03	902
	Philippines	2012	1,086	20.8	1.23	1,085	22.4	1.27	1,086	3.7	0.03	1,086
	Qatar	2010	979	22.2	1.33	979	56.4	1.59	978	4.4	0.02	979
	Singapore	2012	1,710	37.8	1.17	1,710	51.0	1.21	1,710	4.1	0.02	1,710
	South Korea	2010	1,126	26.6	1.32	1,122	33.4	1.41	1,124	4.0	0.02	1,116
	Thailand	2013	1,171	39.9	1.44	1,165	21.3	1.22	1,132	4.1	0.02	1,171
	Turkey	2011	1,470	25.6	1.14	1,462	28.6	1.18	1,470	3.9	0.02	1,436
	Uzbekistan	2011	1,351	26.7	1.21	1,328	22.0	1.13	1,351	3.9	0.02	1,350
	Viet Nam	2006	1,317	37.4	1.34	1,299	16.6	1.03	1,316	3.6	0.02	1,317
	Yemen	2014	915	14.8	1.19	894	16.7	1.23	915	3.9	0.03	915
Europe	Albania	2008	1,406	60.7	1.33	1,340	28.6	1.21	1,394	3.7	0.02	1,389
	Andorra	2005	953	90.0	0.97	951	78.3	1.34	953	4.2	0.02	953
	Austria	2008	1,297	69.4	1.29	1,279	44.0	1.38	1,297	4.1	0.02	1,296
	Belarus	2011	1,345	48.6	1.40	1,277	63.2	1.32	1,345	3.3	0.02	1,328
	Belgium	2009	1,277	82.2	1.07	1,276	48.4	1.40	1,276	4.0	0.02	1,277
	Bosnia	2008	1,372	64.2	1.30	1,359	36.1	1.32	1,332	3.8	0.03	1,369
	Bulgaria	2005	2,099	58.0	1.10	2,022	50.5	1.09	2,096	3.6	0.02	2,094
	Croatia	2008	1,233	83.9	1.06	1,193	49.8	1.43	1,219	3.7	0.03	1,227
	Czech Republic	2008	1,440	59.9	1.31	1,403	56.3	1.32	1,419	3.8	0.02	1,437
	Denmark	2008	1,290	97.0	0.48	1,286	60.1	1.37	1,282	4.3	0.02	1,290
	Estonia	2011	1,224	61.2	1.40	1,206	54.7	1.42	1,224	3.6	0.02	1,221
	Finland	2005	1,871	84.0	0.85	1,846	53.2	1.16	1,861	3.8	0.02	1,864
	France	2008	2,037	82.9	0.84	2,033	50.6	1.11	2,034	4.0	0.02	2,036
	Germany	2013	1,677	65.8	1.16	1,670	49.0	1.22	1,670	4.0	0.02	1,676
	Great Britain	2009	2,024	81.5	0.87	1,990	38.3	1.08	2,024	4.0	0.02	2,024
	Greece	2008	1,178	63.7	1.41	1,164	35.6	1.40	1,177	4.2	0.02	1,177
	Hungary	2008	2,180	76.5	0.91	2,164	50.7	1.07	2,179	3.7	0.02	2,180
	Iceland	2009	715	97.6	0.57	710	58.8	1.84	714	4.2	0.04	711
	Ireland	2008	838	73.5	1.55	810	45.0	1.74	822	4.4	0.03	829
	Italy	2005	2,183	66.3	1.03	2,115	36.8	1.05	2,128	3.9	0.02	2,177
	Latvia	2008	1,241	71.7	1.31	1,181	60.4	1.39	1,239	3.4	0.02	1,238
	Lithuania	2008	1,221	64.2	1.39	1,188	57.0	1.42	1,219	3.5	0.02	1,218
	Luxembourg	2008	1,377	78.4	1.11	1,369	51.3	1.35	1,373	4.1	0.02	1,376
	Macedonia	2008	1,371	52.8	1.37	1,337	38.2	1.32	1,358	4.0	0.03	1,367
	Malta	2008	1,180	59.7	1.44	1,164	35.8	1.40	1,179	3.9	0.02	1,180
	Moldova	2006	2,225	37.6	1.04	2,155	35.5	1.02	2,223	3.4	0.02	2,203
	Montenegro	2008	1,361	75.2	1.19	1,320	42.8	1.35	1,346	3.7	0.03	1,357
	Netherla	2012	1,553	78.5	1.07	1,490	38.8	1.24	1,538	3.9	0.02	1,543
	Norway	2008	1,854	92.4	0.62	1,849	60.5	1.14	1,847	4.2	0.02	1,854
	Poland	2012	819	56.8	1.74	808	47.0	1.75	813	3.8	0.03	819
	Portugal	2008	1,148	66.3	1.40	1,138	54.9	1.47	1,142	3.6	0.02	1,148
	Romania	2012	1,273	38.4	1.38	1,245	39.2	1.38	1,261	3.7	0.02	1,272
	Russia	2011	2,121	39.0	1.07	2,079	59.1	1.08	2,090	3.4	0.02	2,106
	Serbia	2008	1,319	71.4	1.27	1,265	39.0	1.36	1,296	3.6	0.03	1,309
	Slovakia	2008	1,190	55.7	1.47	1,148	51.6	1.45	1,189	3.6	0.03	1,184
	Slovenia	2011	878	80.2	1.35	877	53.5	1.69	873	3.9	0.03	878
	Spain	2011	971	85.5	1.14	948	39.9	1.57	970	4.0	0.02	969
	Sweden	2011	938	95.2	0.70	934	54.1	1.63	934	4.1	0.03	937
	Switzerland	2007	2,071	72.0	0.99	2,055	50.6	1.10	2,059	4.2	0.02	2,069
	Ukraine	2011	1,218	50.2	1.47	1,151	47.0	1.43	1,218	3.3	0.02	1,206
Oceania	Australia	2012	1,185	75.7	1.25	1,174	42.0	1.43	1,184	4.1	0.02	1,182
	New Zealand	2011	666	82.4	1.49	653	56.5	1.93	660	4.2	0.03	659

*SEM, Standard error of the mean.*

Across the sample of 97 countries, PEN ranged from to 97.6% in Iceland to 7.8% in Egypt. PEN were held by more than 90% of respondents in five countries (Iceland, Denmark, Sweden, Norway, Andorra) and more than 60% of respondents in 42 countries. However less than 50% of respondents reported PEN in 43 countries, less than 20% did in 14 countries, and in two countries (Egypt and Azerbaijan) less than 10% of respondents reported PEN. We found gender differences in PEN in the majority of countries: in 94 countries more women compared to men reported PEN, with the biggest gaps in Lithuania (33.1 percentage points (%-points) higher among women) and Belarus (32.4%-points).

On average FTE ranged from 78.3% in Andorra to 10.6% in Mali. In 69 out of 97 countries, average FTE was below 50%. In 94 countries, FTE was lower among women compared to men. Average SRH score ranged from 4.45 (out of 5) in Nigeria to 3.13 in Belarus. In 62 out of 97 countries, the average SRH score was between 3.13 and 4 and in 35 countries the average score was 4.0 or more. In 80 countries, SRH was lower among women compared to men.

#### Pro-equality Norms and Full-Time Employment

**FIGURE 1:**
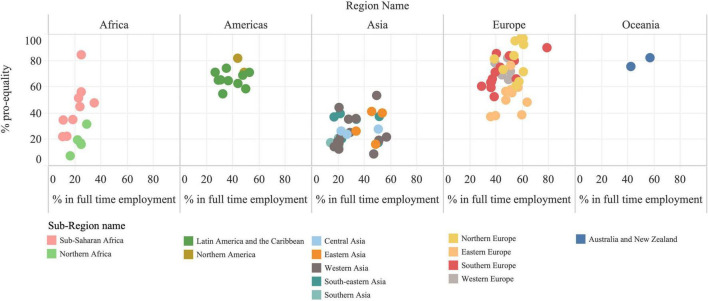
Average pro-equality norms and full-time employment. Each dot represents a country. Countries are grouped by UN regions and colors indicate UN Sub-Regions.

Figure 1 shows country-level PEN and FTE grouped by UN geographical regions and sub-regions (see Supplementary Figure 1 for the sample without regional groupings). Overall, we found a positive correlation between country-level FTE and PEN (correlation coefficient 0.5805, *p* < 0.001), with large between-region variation in both variables. Both PEN and FTE were lowest in Africa and Asia and highest in (particularly Northern) Europe. Countries in Africa and Asia showed consistently < 60% in PEN and FTE, with Ethiopia being the only exception (84.6% PEN). In contrast, most countries in all other regions showed PEN > 60%. FTE varied less by region than PEN. In Africa, FTE ranged from 10.6% in Mali to 34.5% in South Africa. In Asia, the range was 14.0% in India to 56.4% in Qatar. In Europe, FTE ranged from 28.6% in Albania to 78.3% in Andorra. Adjusted regression models using individual-level data for each country showed that FTE was associated with a statistically significant increase in the odds of holding PEN in 32 countries: Adjusted odds ratios (aOR) ranged from 1.18 in Russia to 2.8 in Rwanda. In two countries (Andorra; aOR = 0.45 and Pakistan; aOR = 0.25) FTE was associated with a statistically significant decrease in PEN.

**FIGURE 2:**
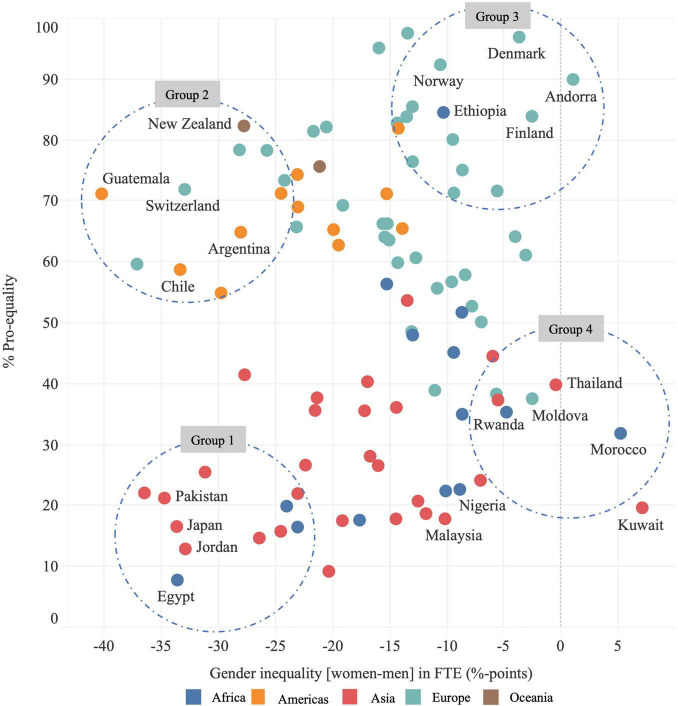
Average pro-equality norms (PEN) and full-time employment (FTE). Each dot represents a country and colors indicate UN Regions. Negative values indicate that FTE is lower among women compared to men. Dotted circles show four country grouping based on levels of PEN and FTE.

Figure 2 shows PEN and gender inequality in FTE. Gender inequality was as large as 40.3%-points in Guatemala (FTE: 73.6% men; 32.3% women) and as small as 0.5%-points in Thailand (FTE: 21.3% men; 20.8% women). In three countries (Andorra, Morocco and Kuwait), FTE was higher by 1–7%-points among women. There was no correlation between country-level PEN and gender inequality in FTE (correlation coefficient 0.1113, *p* = 0.2778).

Figure 2 shows that countries tended to group with other countries in their geographical proximity or with common cultural backgrounds. At the same time, these natural groupings revealed exceptional countries; that is, countries that did not naturally cluster geographically or culturally with the leading group of countries in their geography. We identified four natural groupings.

The first group includes countries with large gender inequalities in FTE and low PEN (e.g., Egypt, Jordan, Pakistan, but also Japan). These are countries where more men than women were in FTE, and where people stated that at times of scarcity men should be given priority to jobs. The second group includes countries with large gender inequality in FTE and high PEN (e.g., Guatemala, Argentina, Chile, but also Switzerland). These are countries where more men than women were in FTE, but where people disagreed that at times of scarcity men should be given priority to jobs. The third includes countries with small or no gender inequality in FTE and low PEN (e.g., Moldova, Thailand, Rwanda, Morocco). These are countries with almost as many women as men in FTE, but where people stated that, at times of scarcity, men should be given priority to jobs. Finally, the fourth group includes countries with small or no gender inequality in FTE and high PEN (e.g., Finland, Denmark, Norway, and Ethiopia). These are countries with almost as many women as men in FTE and where people disagreed that priority should be given to men at times of job scarcity.

### Self-Reported Health, Full-Time Employment and Pro-equality Norms

We then examined SRH and FTE. Average SRH score ranged from 4.45 (out of 5) in Nigeria to 3.13 in Belarus. In 62 out of 97 countries, the average SRH score was between 3.13 and 4 and in 35 countries the average score was 4.0 or more.

Across the sample of 97 countries, there was no overall correlation between SRH and FTE at the country-level (correlation coefficient = −0.0519, *p* = 0.6136). Supplementary Figure 2 shows no large differences in SRH for men and women by quartiles of PEN, and that the range of SRH scores was larger for men compared to women in each quartile of PEN.

**FIGURE 3:**
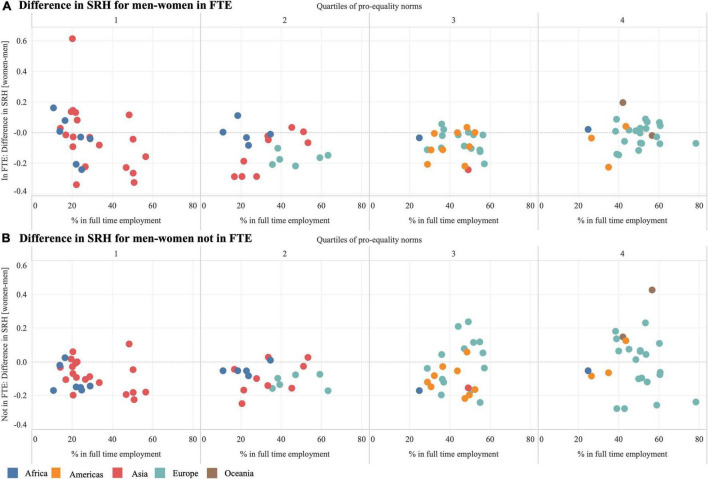
Differences in self-reported health (SRH) for women compared to men among **(A)** those in full-time employment (FTE) and **(B)** those not in FTE. Negative values indicate that SRH is lower among women compared to men. Each dot represents a country and colors indicate UN Regions. Countries are grouped based on quartiles of pro-equality norms.

Figure 3 shows gender inequalities in SRH (men minus women) by FTE status (upper panels: FTE; lower panels: not in FTE) for each quartile of pro-equality view. Negative values indicate that, on average, SRH was lower among women compared to men. In 64 countries, the average SRH score was higher among men in FTE than women in FTE. However, the opposite was true for people living in 33 countries, where the average SRH was higher among women in FTE compared to men in FTE. Roughly two-thirds of these 33 countries were in the lowest quartile of PEN (*n* = 10) or the highest quartile (*n* = 13). Next, we looked at SRH of people who were not in FTE. In 67 countries, the average SRH score was higher for men not in FTE compared to women not in FTE. In the remaining 30 countries, women not in FTE scored higher SRH than men not in FTE; nearly three-fourths of these 30 countries were in the third (*n* = 8) and highest (*n* = 14) quartiles of PEN.

**FIGURE 4:**
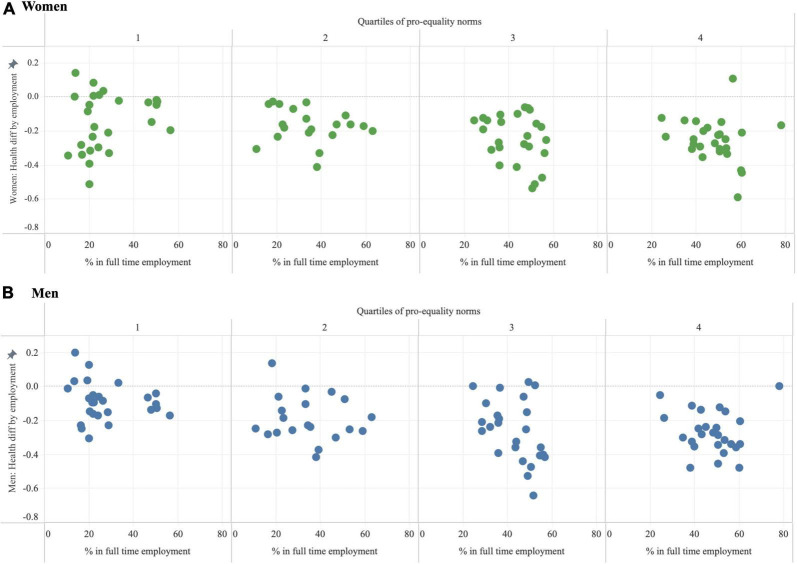
Inequalities in self-reported health (SRH) by full time employment (FTE) among **(A)** women and **(B)** men. Negative values indicate lower self-reported health among those not in full time employment compared to those in full time employment.

Figure 4 shows inequalities in SRH by FTE status among women (upper panels) and men (lower panels). In countries with negative values, SRH was lower among those not in FTE. In 90 countries, women not in FTE had lower SRH than women in FTE. The opposite was true in seven countries. Six of these seven countries where women not in FTE had higher SRH than women in FTE were in the lowest quartile of PEN. Similarly, among men, on average SRH was lower among men not working full time compared to men working full time in 87 countries and higher in ten countries. Five of these ten countries where men not in FTE had higher SRH than men in FTE were in the lowest pro-equality quartile.

### Changes Over Time in Four Selected Country Case Studies

**FIGURE 5:**
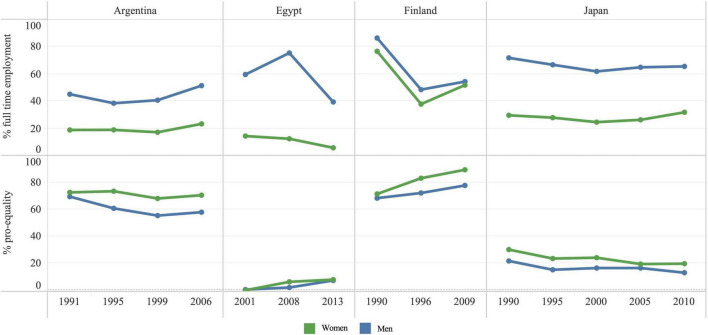
Full-time employment, pro-equality norms, and self-reported health by sex in Argentine, Egypt, Finland, and Japan.

We selected four countries (Finland, Egypt, Japan, and Argentina) as representative case studies for a historical investigation of the conditions and events that may have led to the variations in gender norms and FTE observed in the data (Table 2). Figure 5 reports FTE and PEN for the four case study countries.

**TABLE 2:** Overview of conditions and key historical events potentially affecting full-time employment, pro-equality norms, and self-reported health in four selected countries.

Country	Laws prohibiting discriminating based on gender	Parental leave policies (paid)	External shocks	Political system	Religion (majority)	Time frames
Finland	Equality Act (1986)	-Maternity leave (105 weekdays) -Paternity leave (54 weekdays). -Parental leave (158 weekdays). -Partial childcare leave (partial pay). -Unpaid leave until 3 years.	-Economic Crisis 1990	Parliamentary republic	Christianity (Evangelical Lutheran/Church of Finland).	1990 1996 2009
Egypt	Labour Law 2014	-Maternity only (90 day). -Unpaid leave until child is 2 years old.	-Asian economic crisis in early 2000s. -September 11, 2001 attack -2011 Revolution -2013 coup d’état	Unitary semi-presidential republic	Islam	2001 2008 2013
Japan	Equal Employment Opportunity (1985)	Maternity only (90 days).	-Asian financial crisis -Economic stagnation 1991-2001	Bicameral parliamentary representative democratic constitutional monarchy	Shintoism and Buddhism	1990-2010
Argentina	Labour Law	-Maternity (6 weeks prebirth; 8 weeks post birth) -Paternity leave (7 days). -Childcare Leave up to 1 year (partial pay).	2002 Economic Crisis	Federal presidential constitutional republic	Christianity Catholicism	1991 1995 1999 2006

#### Finland

Finland was the first country to offer women full political rights (Brunila and Kallioniemi, 2018), implementing gender equality legislation as early as the mid 19th century. According to WVS surveys conducted in Finland (1990, 1996, 2009), in 1990 86.5% of men were in FTE compared to 76.8% of women—a gender gap of 9.8%-points (Figure 5). FTE declined by 1996 to 48.5% for men and 38.0% for women. Although FTE increased by 2009 to 52.0% for women and 54.5% for men, it was still lower than 1990 levels. However, by 2009, the gender gap in FTE had declined to 2.6%-points. PEN improved among men and women at each time point and by 2009, 89% of women and 78.0% of men expressed pro-equality views about women’s access to jobs during times of scarcity.

Finland experienced its own economic crisis in 1990 resulting in a 14% GDP drop by 1993. Unemployment increased from 3% in 1990 to 20% in 1994 (Honkapohja and Koskela, 1999). Although GDP recovery started in 1993, unemployment rates continued to be high throughout the 2000s. In fact, the effect of job loss for men during the recession is viewed as a contributor to the decline in gender wage gap in the country (Kyyrä and Pesola, 2018).

Finland began to introduce paternity leave policies in the 1970s, which were officially extended to three weeks in 1991 (Eerola et al., 2019). This may have played a role in shifting fathers’ roles in parenting and childcare responsibilities, which in turn might help explain the fall in FTE for men. One would expect the increase in paternity leave to also result in an increase in FTE for women, which is not shown in our data; however, paternity leave of relatively short duration (e.g., <4 weeks) is usually taken right after the baby is born while the mother is also on leave. The lack of increase of women’s participation in FTE is suggestive of the fact that other macro factors might have been at play in holding women’s participation back. The early 1990s were also important for women’s rise to leadership positions in both the private and public sectors (Virtanen, 2012). In 1990 children also obtained the subjective right to municipal day care until age 3, which was expanded to include children until school age in 1996. The 2000s brought on more reforms and changes including more women gaining power. In 2000, Finland elected its first woman president, in 2006 the first woman Chief of Justice of the Supreme Court, and in 2010 the first female in the Evangelical Lutheran Church was appointed (Kautto et al., 2001; Centre for Gender Equality Information, and Finnish Institute for Health and Welfare, n.d.). The 2000s also brought on reforms of the Equality Act. In the 2010s, the Equal Marriage Act and Maternity Act were passed. Hence, mothers in Finland were provided with maternity grants, leaves and allowances and fathers were allowed paternity leaves and either parent could also apply for an unpaid parental leave for up to three years (Kautto et al., 2001; Centre for Gender Equality Information, and Finnish Institute for Health and Welfare, n.d.). Marriage continues to be the most supported family form with an average two-child family size (Jalovaara and Fasang, 2020).

These factors may have helped to account for the rise in PEN and the closing of the gender gap in jobs, while the ongoing effect of the 1990 economic crisis may provide an explanation on why FTE was lower for women as well as men in 2009 compared to 1990.

Although Finland now has a small gender gap in FTE, earnings continue to differ according to gender (World Economic Forum, 2018), possibly partly because women are more likely to take parental leave, which could result in a slower salary increase in the career. The labor market remains gender segregated with women taking on lower-paying jobs and taking longer parental leaves (Emerek, 2008; Kosonen, 2014). This may be due to the persistence of traditional gender norms. A study in 2020 looking at gender differences in early career choices among dentists reported that women perceived themselves as comforters while men viewed themselves more as technicians. Cultural ideals related to appropriate jobs for men and women might have oriented their career choices (Karaharju-Suvanto et al., 2021). This showed that even in dentistry, a field traditionally dominated by females in Finland, male graduates seemed to cluster in the private sector, choosing more financially rewarding specialties that required technical skills, while women tended to favor the public sector and leaned toward specialties that focused on social care and health promotion (Karaharju-Suvanto et al., 2021).

Another study which focused on assessing equality within education in Finland identified several persistent barriers including hierarchical gender order and heteronormativity that were expressed through student essays and reflected teacher attitudes (Brunila and Kallioniemi, 2018).

#### Egypt

Gender equality in Egypt has been recently receiving more attention due to the political movements in this and other Arab states (Sorbera, 2014). Although most Arab governments are committed under the Committee on the Elimination of Discrimination Against Women to promote gender equality, in 2015 Egypt ranked 136th out of 145 countries in the Gender Gap Index of the World Economic Forum (Abbott, 2017). WVS surveys in Egypt (2001, 2008, 2013) show that in 2001, FTE among men was 59.7% compared to 14.5% among women with a gender gap of 45.2%-points (Figure 5). Although the FTE gender gap decreased to 33.7%-points in 2013, gender inequalities remained large. Pro-equality norms were lower than 1% among both men and women in 2001 and increased slightly to 8.1% among women and 7.4% among men by 2013.

The feminist movements in Egypt are well documented (Ramdani, 2013). Two historical waves of feminism took place in the country: the first in the 1920s–1930s, when the Egyptian Feminist Union was found by Huda Shaarawi, and the second in the 1950s when, under the leadership of Doria Shafik, a powerful movement demanding equal pay for equal work was born. In the 1970s and 1980s, Egypt worked to narrow the gender gap in education, which led to a new generation of educated women that nevertheless remained largely unemployed (Assaad et al., 2000).

In the early 2000s, in response to a surge in Egyptian scholars calling for more traditional roles for women, a new Islamic feminist movement emerged (Rock, 2010). This new movement achieved important milestones, including women’s right to divorce. Later, the Arab Spring movements and the Egyptian revolution in 2011 further contributed to bringing women’s rights back onto the civil and political agendas, especially due to the role that women played in these movements (Khodair and Hassib, 2015). We observe in the data a timid, yet consistent, rise in PEN, possibly related to the greater mediatic and political visibility that women gained during this time.

Today, in general, women in Egypt continue to adhere to gender norms that assign them social status based on their capacity to take care of their household and children (Barsoum, 2019). Work opportunities for women are constrained by low pay, long hours, and gender norms regulating access to heritage and property. Married women’s access to FTE is hampered by the perceived impossibility of reconciling family and work obligations (Barsoum, 2019). The introduction of policies that aimed to improve women’s working conditions, notably generous maternity leave with no corresponding paternity leave, raised the cost of hiring women and acted as a barrier to women’s employment (Assaad et al., 2000). These policies, coupled with a hostile normative environment, might partly explain why women’s FTE dropped slightly between 2001 and 2008, while men’s FTE increased almost 20%. However, macroeconomic shocks have had a great impact on Egypt’s economy and unemployment rates. Egypt in the late 1990s was experiencing growth in its economy after a few years of decline (Malec et al., 2016). Yet in the early 2000s, the Asian crisis and aftermath of September 11 terror attacks provided external shocks to the country (Malec et al., 2016). From 2003-2008, there was a gradual growth in the economy, with GDP growth increasing from 2.4 to 7% (Assaad and Krafft, 2015, 29). Yet, in 2008, the global financial crisis hit, and had a big impact on Egypt’s tourism industry, Suez canal, oil trade, GDP, and employment (Mansour, 2011). The period between 2008 and 2011 was one of increased lay-offs, high unemployment rates and rising irregular employment (Mansour, 2011). In 2011, the Egyptian revolution erupted and focused on socio-political and economic issues including political freedom and rising unemployment rates. These economic shocks are reflected in downturns in men’s and women’s FTE in 2013.

#### Japan

Japan ranked 110th out of 149 countries according to the 2018 Gender Gap Index, and had the second largest gender wage gap among Organisation for Economic Co-operation and Development nations (37 nations) in 2018 (24.5%) (Murakami and Borgonovi, 2018). WVS surveys for Japan (five surveys between 1990 and 2010) show that in 1990, FTE was 72.0% among men and 29.8% among women with a gender gap of 42.2%-points (Figure 5). By 2010, FTE among men was 65.7 and 32.0% among women; thus, a large gender gap (33.7%-points) remained. Pro-equality norms were low for both men (22.0%) and women (30.3%) in 1990 with small downward trends to 13.2% of men and 20.0% of women in 2010.

Japan has two legal categories of workers: regulars and non-regulars (Piotrowski et al., 2015; Yamaguchi, 2019). Regular workers have indefinite terms with no specific job obligations and are protected from lay-offs, while non-regular workers have fixed-term contracts, lower wages and lower increases in salaries irrespective of age and gender. Because young graduates are favored for regular work, women who leave the job for childbearing and try to re-enter are rarely hired as regulars, losing the opportunity to have the benefits that come with this recognition. More than half of women aged 20-65 are non-regular compared to 14.1% of men of the same ages (Piotrowski et al., 2015; Yamaguchi, 2019).

This disadvantage may explain in part why the FTE gap between men and women has narrowed very little in the 20 years covered by the WVS surveys. The gap does not appear to stem from the level of education of women compared to men. The 1985 Equal Employment Opportunity Law was passed to protect women’s rights in the workplace and educational attainment is essentially equivalent for men and women. Policies regarding child care appear to reinforce gender-stereotypical roles. Mothers can apply for maternity leave from 6 weeks prior to eight weeks after childbirth. If a child cannot be placed in childcare, this leave may be extended up to when the child is 18 months old. While fathers can also apply for childcare leave with the leave starting any day after the child’s birth, they are only eligible for a total of seven days while their wife is on maternity leave. Women can also take two 30-min breaks during work hours to breastfeed for the first year of the child’s life (Asai, 2015). Birth allowance, medical services for children and sick leave for child care is available for both parents. However, women are disadvantaged by the gender norms assigning to them the role of primary caretaker at home, since job promotions are given as rewards to employees who work extended hours (Osawa, 2015; Coleman, 2016; Yamaguchi, 2019).

PEN have remained fairly stable at low levels over the same time period, despite active feminist movements in Japan. In 1919, the New Woman Association achieved important civil successes for women, including granting them the right to join political parties and participate in political events, as well as having equitable divorce laws (Molony, 2000). Women gained the right to vote after the second World War. Resistance to norm change remains strong, however, as suggested by the negative depiction of women’s rights movements in Japanese media (Molony, 2000).

#### Argentina

WVS surveys in Argentina (1991, 1995, 1999, 2006) showed that in 1991, FTE was 45.3% among men and 19.0% among women with a gender gap of 26.3%-points (Figure 5). Between 1991 and 2006 there were small increases in FTE for men and women and by 2006, FTE had increased to 51.5% among men and 23.4% among women, thus slightly widening the gender gap to 28.1%-points. The majority of men and women held pro-equality views and PEN were higher among women compared to men at all time points. By 2006, there was a small decline in pro-equality norms which were held by 70.7% of women and 58.1% of men.

Feminist movements in Argentina in the 1960s and 70s were strongly influenced by Marxist philosophy (Pite, 2014), to the point that they often resorted to strikes as a means to obtain civil working rights. In the 1970s, these strategies led toincreased female labor participation and women’s educational attainment. Partially because of their Marxist footprint, after the 1976 coup, the new right-wing government distrusted feminist movements as subversive to the state. At the same time, the left wing disapproved of their intellectual stances, ostracizing them as a group of *petit bourgeois* (Veleda da Silva and Lan, 2007). In the 1990s, after democracy was regained, feminists embraced a human rights approach, creating key alliances with political parties and international organizations as well as local activist groups. The rise of neoliberalism in the 1990s led to a further opening up of the Argentinian economy including privatization and decreases in public funding. This was accompanied with a rise in unemployment, noticeable in a slight decline in the percentage of men in FTE (Figure 5; Pite, 2014).

The drop in employment rates led to recession and devaluation of the currency, and eventually to the 2001 economic crisis (Iturriza et al., 2011). Unemployment rose from 15.1 to 20% between 2000 and 2002 and 63% of households experienced declines in income by at least 20% (Iturriza et al., 2011). In 2003, Argentina experienced a steady recovery, reaching GDP/capita of $13,440 in 2011. A governmental unemployment assistance plan was launched in response to the economic crisis (Plan Jefas y Jefes de Hogar Desocupados - Program Unemployed Heads of Households), which provided monthly monetary transfer for eligible unemployed household heads for an unlimited duration (Iturriza et al., 2011).

As the employment rates fell, so did the PEN. We hypothesize that scarcity of jobs, together with a widespread malcontent due to the perceived failed neo-liberal democratic endeavor, could have contributed to tightening of traditional gender norms. The fall in PEN was greater for men than for women, despite the fact that men had higher FTE throughout the decade (Figure 5). As employment started to rise again for both men and women, so did PEN. SRH also increased in this time period. While the rise in unemployment had adverse effects on health (and specifically maternal and infant health) (Wehby et al., 2017), our data suggest that a rise in employment was associated with better living conditions for all in the country, as the SRH of all, independent of their FTE status, increased again in the last wave of data. In a hostile normative environment, women always had and continue to have higher numbers of lower-paying jobs, lagging in leadership positions in both private and public sectors. Gender norms (a macho culture) have been found to be one of the most critical factors in holding back women’s potential (Barrancos, 2006). Institutional factors also contributed to women’s reduced FTE, including a short maternity leave period (90 days with an extra 180 unpaid) with no corresponding paternity leave (Maternity Leave and Benefits - Argentina, 2021).

## Discussion

In this paper, we set out on an investigation to generate hypotheses on the relationship between FTE, PEN, and SRH. Despite the fact that we found a positive correlation between country-level FTE and PEN, we are unable to infer that either gender equality leads to FTE or FTE leads to greater gender equality. There exist several criticisms advanced by culturally relativistic intellectuals on how gender equality is conceptualized and measured by western scholars and international agencies. One such criticism is that the ideal end state envisioned as part of the gender equality movement is strongly biased by the Western (and more specifically Scandinavian) model as one that is globally viable (Nygren et al., 2018). The correlation between FTE and PEN might thus be endogenous, in that industrialized Western countries would inevitably score higher on measures designed to match worldviews that are more common in those countries. Unfortunately, such speculations are difficult to resolve. We lack consensus on what outcomes are signs of gender equality (Kabeer and Natali, 2013) and scholars of gender equality face continuously the intricacies of applying a universal definition of gender equality (provided we had one) across different cultural and social settings (Raj et al., 2019; Jayachandran, 2020; Ortiz et al., 2020).

Research on how SRH differs based on employment status is limited. Our findings about inequalities in SRH by gender and FTE suggest that, on average, SRH scores are lower among women employed full time compared to men, and that SRH scores are lower among women not in FTE compared to women who are working full time. More specifically, we found that, in about two-thirds (64 of 97) of the countries in our sample, men benefit (in terms of SRH) from being in FTE more than women. While this confirms what has been reported elsewhere in the literature (Ellison, 1999; Åhs and Westerling, 2006; Campos-Serna et al., 2013; Au and Johnston, 2014; Borgonovi and Pokropek, 2016; Ross, 2017; Esteban-Gonzalo et al., 2018) it is remarkable to notice that the opposite was true in the remaining one third (*n* = 33) of countries. Similarly, being in FTE was beneficial for both men and women as compared to people of the same sex not in FTE. However, again, the advantage of being in FTE was observed in the large majority of the countries in our sample, but not in their totality. We hypothesize that the very normative beliefs that make it appropriate for a man or a woman to be in FTE could also contribute to the creation ofstructural and material conditions where it can be beneficial, for certain people and in certain cases, not to be in FTE, depending on other associated factors. For instance, in a given country where most women work inside the household, a woman in FTE might be frowned upon and ostracized in ways that would impact her social life and mental health, eventually affecting how she assesses her SRH. In the same country, a man working full time might have to work extra hours because his wife is not allowed to work, deteriorating his SRH. The opposite is also entire possible; that is, in a country where it’s a sign of success and agency for women to work full time, those who don’t might experience a feeling of isolation that reduces their SRH. Our findings point to the need for further research to understand not only how employment brings material benefits that are likely to improve one person’s SRH (e.g., economic independence, health coverage, social connections), but also how counter-normative employment patterns may affect people’s health and wellbeing, and how effective policy-makers can integrate in their work purposeful actions to counter-act the negative effect of not complying with the existing system of norms prescribing or proscribing FTE.

Our findings also show that, in most countries, respondents believed that in times of scarcity jobs should go to men first. At these challenging times marked by the COVID-19 pandemic, where job scarcity is a reality faced by many, women may be facing additional challenges accessing FTE because of similar gender norms. As governments strive to increase people’s health and access to employment, policy makers should thus take into account how gender norms can reduce women’s access to FTE, and potentially their health, during similar breakdowns of the social or economic *status quo*. Our findings contribute to prior research underscoring the importance of gender-responsive workplace polices, working conditions, and pay equality in improving the participation and experiences of women in the labor force (Baxter and Wright, 2000; Benschop, 2009; Durbin and Tomlinson, 2010; Alkadry and Tower, 2011; Alkadry and Tower, 2006; Zeng, 2011; Xiu and Gunderson, 2014).

Our study has some important limitations. A single measure of FTE didn’t allow us to compare how the same job resulted in differences in salary, job security or job conditions, limiting our understanding of how FTE can affect women’s and men’s SRH. The measure of FTE could be hard to compare between countries, for instance due to differences in the informal job market, in ways that could affect SRH differently. Also, the employement category, self-employed, was subject to wide variation in definition and likely interpretation across countries, and thus could not be used reliably. It is likely that some self-employed individuals work full-time, and thus, our descriptions of FTE may be incomplete to a variable, undefined extent from country-to-country. Our measure of PEN does not enable generalization about the level of overall within-country support for gender equality. Gender equality attitudes vary within individuals, so that, for example, one person can have a pro-equality attitude toward access to employment while holding a double standard toward men and women having premarital sex. Thus, while PEN cannot be held as a representative measure of gender equality broadly, we found it reflected well a specific relation with gender equality in regard to participation in paid employment. Because of limitations in statistical power, as well as the variables available in the surveys, we could not complete an intersectional analysis of how gender interacts with other categories of social discrimination (e.g., ethnicity, immigrant status, socioeconomic status, disability) that would have helped uncover deeper dynamics of social disadvantage and their effect on people’s SRH. Limitations in sample size in survey data commonly prevent intersectional analysis, and require concerted attention (Weber et al., 2019). While country case study selection represented quartiles of PEN and examined employment status and SRH across quartiles, selection was also guided in part by data availability, which limited the countries that could be considered for deeper qualitative analysis. SRH in itself as a variable has been widely criticized (Cislaghi and Cislaghi, 2019). Despite the variable being often used as a satisfactory indicator of people’s general internal sense of wellbeing, it offers little in terms of people’s actual medical conditions. We also cannot evaluate fully the extent to which the fact that the survey was translated in a variety of languages might have impacted participants’ responses. In Arabic, for instance, the only option available for those caring for the home is housewife, which could obviously have affected men’s readiness to declare themselves as such. Despite these limitations, our study contributes to the wider research on how gender norms can affect health and employment.

Focusing on access to employment had two advantages. First, it allowed us to utilize a sex-disaggregated data set containing multiple variables at multiple times across nearly 100 countries. Second it offered an opportunity to engage in a critical reflection on the extent to which access to employment contributes to people’s wellbeing. We could discuss whether FTE (in absolute terms, that is, not equal access by gender) should, in fact, be considered a desirable outcome in gender equality and social justice terms, or whether its promotion hides unwitting biases toward replication of the post-industrial economic order that high income countries have exported through promotion of a capitalist global market (Pontusson, 2005). One might wonder whether FTE is preferable (both in economic and social terms, for instance, to the family care of children and elderly. In our case studies, we offered researchers and policy-makers strategies to make sense of how the historical and material conditions of a given country can affect the relation between women’s FTE and people’s acceptance of it. Our paper is intended to generate critical hypotheses of how SRH, FTE and gender norms interact. Our intent is to open new avenues for the critical investigation of this important intersection, as we work to achieve greater equality in the labor market and, more generally, across all aspects of people’s lives. Future research should identify and measure additional variables which reflect gender norms related to employment, and clarify and expand data on employment status.

## Conclusion

In this paper we set up an investigation to understand the relation between gender norms, FTE for men and women, and SRH in 97 countries. We found that gender norms play a key role in determining women’s participation in FTE across different cultural contexts. However, the relation between gender norms and women’s participation in FTE is heterogeneous. In two country groups we found the expected relation: gender norms favoring women’s participation in FTE were associated with greater participation in FTE and, vice versa, gender norms against women’s access to FTE were associated with lower participation for women. We observed a mismatch in countries in Eastern Europe and South America. Equitable gender norms in South America did not match representation in FTE (where men were in higher proportion). In countries in Eastern Europe we found gender norms against equal access and small differences in FTE. We hypothesized that these differences in the role played by gender norms are affected by the system of laws and policies that create favorable or unfavorable structural conditions for women to participate in FTE. Across the whole dataset we also found that FTE was associated with higher SRH but, once again, this association varied across normative contexts. For instance, we found that to be a woman in FTE was not associated with greater SRH in Middle Eastern countries. Finally, observing time trends, we found a general movement toward greater equality in both gender norms and participation in FTE. Despite a general movement toward greater gender equality in the world, however, our analysis of selected case study countries shows that political and economic events can generate profound challenges to a seemingly natural progress toward equality. This paper offers both heuristic and methodological avenues for future research and policy. Through the use of FTE and SRH as a case study of gender norms, we created a method to theorize ecologically how gender norms affect people’s choices and actions, and ultimately their health. We also found that policies that exclusively target the practice of interest (in this case FTE) are necessary but not sufficient to improve people’s health. As policy-makers design purposeful interventions to improve their economies and the quality of life for men and women living in their countries, while striving to improve their access and participation in FTE, they should also work to generate greater acceptance for those men and women that do decide to participate in FTE, independently of what others are doing in their cultural context.

## Data Availability Statement

The original contributions presented in the study are included in the article/Supplementary Material; further inquiries can be directed to the corresponding author.

## Author Contributions

BC conceptualized the study, reviewed all analyses, drafted and revised the manuscript. AB conducted quantitative analyses and contributed to drafting the manuscript. EH constructed and cleaned the dataset, conducted preliminary analyses and reviewed all drafts of the manuscript. NH conducted case study analyses and contributed to drafting the manuscript. AW conceptualized the study and reviewed all drafts of the manuscript. GD conceptualized the study, reviewed all analyses, and reviewed all drafts of the manuscript. All authors reviewed drafts of the manuscript and approved the final version.

## Conflict of Interest

The authors declare that the research was conducted in the absence of any commercial or financial relationships that could be construed as a potential conflict of interest.

## Publisher’s Note

All claims expressed in this article are solely those of the authors and do not necessarily represent those of their affiliated organizations, or those of the publisher, the editors and the reviewers. Any product that may be evaluated in this article, or claim that may be made by its manufacturer, is not guaranteed or endorsed by the publisher.
