# Supplementary material for: Gender Norms and Gender Equality in Full-Time Employment and Health: A 97-Country Analysis of the World Values Survey
Source: Front Psychol. 2022 May 31;13:689815. doi: 10.3389/fpsyg.2022.689815 (PMC9234689; doi:10.3389/fpsyg.2022.689815)

Supplementary Material

Gender norms and gender equality in full-time employment and health: A 97-country analysis of the World Values Survey

Beniamino Cislaghi[1*], Amiya Bhatia[1], Emma Sofia Thonander Hallgren,[2] Nour Horanieh[1], Ann M. Weber,[3] Gary L. Darmstadt[4]

[1]Department of Global Health and Development, London School of Hygiene and Tropical Medicine, London, UK

[2]Center for Health Sciences, Stanford University School of Medicine, Palo Alto, CA, USA

[3] School of Community Health Sciences, University of Nevada, Reno, NV, USA School of Community Health Sciences, University of Nevada, Reno, NV, USA

[4]Department of Pediartrics, Stanford University School of Medicine, Stanford, CA, USA

Supplementary Tables

Supplementary Table S1. Sample characteristics by country

Supplementary Figures

Figure S1. Pro-equality norms and full-time employment in 97 countries

Figure S2: Self-reported health among men and women by full time employment for quartiles of pro-equality norms

S1. Sample characteristics by country

Country	Age		Education						Employment status															
			Lower		Middle		Upper		Full time		Part time		Self employed		Retired		Housewife		Students		Unemployed		Other	
	Mean	Median	%	n	%	n	%	n	%	n	%	n	%	n	%	n	%	n	%	n	%	n	%	n
Albania	41.8	43.0	40.5	555	41	562	18.6	255	28.6	399	5.3	74	24.1	336	8.8	123	7.6	106	5.5	77	18.3	255	1.7	24
Algeria	37.5	35.0	42.7	393	32.4	298	24.9	229	21.9	235	12.9	139	10	108	4.1	44	23.4	252	13.7	147	13.4	144	0.6	6
Andorra	40.1	38.0	22.2	210	44.1	418	33.8	320	78.3	746	3.3	31	11.9	113	2.2	21	1.9	18	1.4	13	0.7	7	0.4	4
Azerbaijan	40.8	41.0	2.8	25	57.3	516	39.9	359	46.7	422	9.1	82	8.1	73	5.3	48	15.2	137	2.1	19	13.5	122	0	0
Argentina	41.2	39.0	47.3	398	35.4	298	17.2	145	36.3	309	13.2	112	13.5	115	7.1	60	19.3	164	3.8	32	6.3	54	0.6	5
Australia	48.0	50.0	17.3	356	27.4	565	55.3	1139	42.5	1003	17.5	413	10.8	255	17.3	409	6.3	148	1.7	41	2.7	63	1.2	28
Austria	43.4	43.0	13.7	178	75.4	977	10.9	141	44	571	11.1	144	6.8	88	17.6	228	7.6	98	8.5	110	2.5	32	2	26
Bahrain	40.1	40.0	33.6	370	40.1	441	26.3	289	19.4	223	27	310	16.7	192	7.7	88	16.3	188	3.7	42	9.3	107	0	0
Armenia	42.0	42.0	7.8	165	60.2	1281	32	681	27.1	577	8.1	172	8.3	176	8.5	182	24.7	527	3.1	66	19	405	1.2	26
Belgium	44.4	45.0	26.8	342	37.7	481	35.5	453	48.4	618	9.6	123	4.4	56	13.1	167	6.4	82	6.1	78	7.6	97	4.3	55
Bosnia	40.3	38.0	21.5	292	65.2	886	13.3	181	36.1	481	2.8	37	3.4	45	9.8	130	10.2	136	9.2	122	27.7	369	0.9	12
Brazil	40.7	39.0	50.6	1325	32.3	847	17.1	448	32.6	858	6.7	176	16	422	11.6	305	15.4	405	2.6	68	14.5	383	0.6	16
Bulgaria	45.9	46.0	21.1	432	56.6	1161	22.3	457	50.5	1058	3.5	74	4.2	87	24.3	509	3.3	70	2	41	11.7	245	0.6	12
Belarus	41.8	41.0	2.9	77	57.6	1530	39.5	1049	63.1	1677	7.7	205	3.4	90	13.8	366	4.2	111	4.4	118	2.7	73	0.6	17
Canada	44.7	45.0	19.4	338	49.6	863	30.9	538	43.5	760	11.9	209	7.1	125	14.6	256	7	123	3	53	11	193	1.7	30
Chile	42.1	42.0	29.4	508	51.1	883	19.5	336	43.8	767	8.9	155	7.8	137	5.7	100	21.9	383	7.3	127	4.6	81	0	0
China	43.9	43.0	29.1	991	56.4	1922	14.5	495	57.1	2227	15.1	587	3.5	137	7	274	7.8	305	2.4	95	3.3	128	3.7	144
Colombia	40.4	39.0	30.1	387	42.1	541	27.8	358	28.7	384	6.5	87	22.1	295	3.4	46	15.9	213	2.5	34	14.6	195	6.2	83
Croatia	41.8	42.0	16.4	202	61.9	760	21.7	266	49.8	607	2.9	35	3.1	38	16.3	199	4.3	52	7.2	88	15.7	191	0.7	9
Cyprus	41.5	41.0	24.1	626	46.3	1204	29.6	768	49.2	1289	4.5	119	9.6	252	9.5	249	14.9	391	5.8	153	5.3	140	1	25
Czech Rep.	45.2	46.0	8.8	127	78.2	1123	13	186	56.3	799	1.9	27	6.2	88	19	269	3.9	55	3.7	52	4.3	61	4.8	68
Denmark	46.2	46.0	20.3	259	41.1	523	38.6	491	60.1	771	7.7	99	6.5	83	16.7	214	0.5	6	4.8	62	2.1	27	1.6	20
Ecuador	39.7	38.0	41	429	30.5	319	28.6	299	32.1	340	7.5	79	25.4	269	2.4	25	22.6	239	4.3	45	5.8	61	0	0
Ethiopia	31.3	28.0	45.3	537	40	474	14.7	174	24.7	333	4.5	61	21.6	292	3.4	46	11.6	156	11.6	156	22.1	298	0.5	7
Estonia	44.7	45.0	25.2	610	44.2	1071	30.6	740	58.1	1407	7.2	175	5.3	129	14.1	341	5	120	3.6	87	5.5	133	1.2	28
Finland	44.9	45.0	16.2	300	39.3	727	44.5	824	53.2	990	5.1	94	5.6	104	17.5	325	2.8	53	5.3	98	7.6	142	3	55
France	44.1	44.0	28.8	584	38.8	788	32.4	658	50.6	1029	7.7	156	4.6	94	16.2	330	8	163	2.9	59	6.9	141	3	62
Georgia	42.4	41.0	3.7	132	57.2	2048	39.1	1401	20.1	721	4.9	176	13.2	472	9.9	356	14.8	529	2.1	75	34.6	1240	0.3	11
Palestine	36.7	35.0	26.7	235	32.5	286	40.7	358	21.6	195	7.2	65	13.5	122	3.3	30	35.4	319	8.2	74	10.4	94	0.3	3

Country	Age		Education						Employment status															
			Lower		Middle		Upper		Full time		Part time		Self employed		Retired		Housewife		Students		Unemployed		Other	
	Mean	Median	%	n	%	n	%	n	%	n	%	n	%	n	%	n	%	n	%	n	%	n	%	n
Germany	46.2	47.0	25	1256	55.2	2772	19.8	995	42.6	2156	11.7	592	4.5	228	19	961	5.6	283	3.4	171	10.9	551	2.4	119
Ghana	33.5	30.0	54.7	1272	34.6	805	10.6	247	18.8	504	4.5	121	42.5	1139	1	28	1.2	31	11.8	316	20.1	538	0.2	6
Greece	44.7	45.0	34.1	402	44	518	21.9	258	35.6	419	3.8	45	17.4	205	15	177	17.8	210	4.7	55	5	59	0.6	7
Guatemala	35.1	33.0	35	314	48.7	436	16.3	146	52.3	473	12.7	115	0	0	2.3	21	24.1	218	3.3	30	5.1	46	0.1	1
Hong Kong	43.3	42.0	22.8	443	55.2	1071	21.9	425	46.1	905	7.4	146	3.7	72	12	235	19.8	388	5.2	102	5.7	111	0.1	2
Hungary	42.7	42.0	20.1	437	61.8	1346	18.2	396	50.7	1105	3.1	67	5	109	18.4	401	2.3	51	4.7	103	10.4	227	5.3	116
Iceland	43.0	44.0	22.2	156	41.2	289	36.6	257	58.8	420	9.2	66	12.2	87	1.7	12	2.2	16	4.6	33	5.3	38	5.9	42
India	39.3	38.0	40.6	1016	36.2	906	23.2	581	20.8	632	12.5	381	18	546	2.5	77	12	365	19.6	594	8.9	271	5.6	170
Indonesia	37.2	35.0	20.6	372	44.2	800	35.2	638	33.5	614	12.4	228	18.1	331	2.4	44	16.9	310	10.5	192	6.1	112	0.1	2
Iran	33.8	31.0	35	754	36.5	785	28.5	613	20.2	466	6.7	155	17.2	396	3.6	83	31.3	720	6.6	152	13.9	319	0.5	12
Iraq	37.0	35.0	48.6	1498	29.1	898	22.2	685	18	639	9.5	338	15.2	541	3.8	135	40.1	1426	5.1	183	7.8	277	0.5	16
Ireland	42.5	41.0	37.9	314	39.4	326	22.7	188	45	370	10.9	90	6.1	50	4.6	38	21	173	2.8	23	7.5	62	1.9	16
Italy	43.8	44.0	31.6	678	45.9	986	22.5	483	36.8	783	7.7	164	16.6	354	13.6	289	9.7	206	6.3	134	7.4	157	1.9	41
Japan	46.8	47.0	8.7	257	64.9	1918	26.4	779	46.6	1352	15.5	450	9.8	284	6.6	191	15.6	453	1.6	45	2.3	67	2.1	61
Kazakhstan	39.0	37.0	4.4	59	56.9	765	38.7	521	50.2	677	13.8	186	5.5	74	8.3	112	13.9	187	1.6	21	6.4	86	0.4	5
Jordan	39.9	39.0	39.8	400	34	341	26.2	263	20.2	212	5.2	54	8.5	89	11.4	119	45	472	3.9	41	5.4	57	0.4	4
South Korea	41.5	41.0	6.5	150	36.5	837	57	1308	34.5	796	7.5	173	13.2	305	2.7	62	18.4	424	11.6	268	3.7	86	8.5	196
Kuwait	36.3	34.0	11.7	139	44	521	44.3	524	50.7	602	21.1	251	5.4	64	6.7	80	7.6	90	5.6	66	2.1	25	0.8	10
Kyrgyzstan	38.7	38.0	10.3	140	52.6	715	37.1	505	26.4	361	16.8	229	15.2	208	6.8	93	18.1	247	5.9	81	9.9	135	0.9	12
Lebanon	39.5	39.0	22.1	225	34.9	356	43	439	33.2	363	8.6	94	16.2	177	5.9	64	18.1	198	10	109	7.4	81	0.5	6
Latvia	43.4	43.0	14.9	185	58.3	722	26.7	331	60.4	748	4.2	52	3.6	45	11.9	148	6.9	86	3.1	38	7.2	89	2.7	33
Libya	38.3	38.0	29.8	562	32.2	607	38	717	24.6	489	20.4	406	9.7	192	4.6	91	21.4	425	12.1	241	6.5	129	0.7	14
Lithuania	44.3	45.0	15.1	183	42.2	513	42.7	519	57	695	6	73	5.5	67	13	159	4.9	60	4.8	58	5.6	68	3.2	39
Luxembourg	38.2	36.0	30.4	412	40.7	551	28.9	392	51.3	705	7.9	108	3.3	45	8.7	120	9.7	133	14.2	195	3.3	45	1.6	22
Malaysia	38.0	37.0	25.3	533	60.4	1272	14.3	300	49.6	1079	4.6	99	19.5	425	4.3	93	14.8	322	4.6	99	2.5	54	0.1	3
Mali	37.6	35.0	55.5	331	28.7	171	15.8	94	10.6	130	4.9	60	22.8	280	3.8	47	25.8	316	6.9	85	24	294	1.2	15
Malta	47.4	49.0	71.6	845	10.6	125	17.8	210	35.8	422	3.8	45	3.7	44	13.9	164	35.8	422	1.4	16	4.8	57	0.8	9
Mexico	38.0	36.0	33.8	1021	44.5	1344	21.7	657	28.9	908	8.5	266	17.1	537	2.4	74	29.6	930	3.6	114	9.4	296	0.7	22
Moldova	41.8	42.0	11.6	258	67	1487	21.4	475	35.5	790	9.2	205	7.5	167	13.6	303	7.9	176	5.1	114	19.2	426	1.9	42
Montenegro	41.2	40.0	14.9	201	61.1	825	24.1	325	42.8	576	2.2	29	5.6	76	11.7	158	4.6	62	3.9	53	28.2	380	0.9	12
Morocco	37.5	34.5	42.8	399	46.9	438	10.3	96	40.7	940	14.2	328	32.2	743	1	22	5.5	127	2.1	48	2	47	2.3	54

Country	Age		Education						Employment status															
			Lower		Middle		Upper		Full time		Part time		Self employed		Retired		Housewife		Students		Unemployed		Other	
	Mean	Median	%	n	%	n	%	n	%	n	%	n	%	n	%	n	%	n	%	n	%	n	%	n
Netherlands	47.6	49.0	32.7	1166	34.1	1217	33.1	1181	36.9	1322	20.5	733	4.8	172	14.4	517	9.4	337	2.9	103	4.8	173	6.2	223
New Zealand	46.2	47.0	1	14	42.6	597	56.4	790	54.7	783	18.4	264	1.2	17	8	115	8.3	119	2.3	33	5.9	84	1.1	16
Nigeria	32.1	29.0	22.4	323	56.5	816	21.2	306	13.7	219	4.8	76	43.4	693	1.1	18	10.7	170	19.2	306	7.1	113	0.1	1
Norway	44.2	44.0	19.5	360	32.6	601	47.9	884	60.5	1118	9.1	169	8.7	160	8.8	163	1.9	36	6.2	115	1	18	3.7	68
Pakistan	35.0	35.0	50	426	39.8	339	10.2	87	20	222	7.6	84	9.7	108	0.6	7	45	499	7.7	85	3.1	34	6.4	71
Peru	38.5	37.0	30.5	705	46.5	1075	23	533	22.1	524	4.5	107	39.2	930	3.1	73	19.6	466	6.8	162	4.4	105	0.3	6
Philippines	41.8	41.0	33.9	361	35.8	381	30.3	323	22.4	243	11	120	28.5	310	2	22	17.5	190	1.5	16	17	185	0	0
Poland	43.1	44.0	28.1	819	51.3	1496	20.6	599	46.5	1354	4	117	6.1	179	21.5	627	4.6	135	6	176	10.1	293	1.1	33
Portugal	46.0	45.0	70.1	803	18.3	210	11.5	132	54.9	627	3.2	37	3.9	44	16.7	191	6.8	78	2.5	29	10	114	1.9	22
Qatar	38.4	38.0	15.1	142	37.4	351	47.4	445	56.4	552	0.9	9	2.2	22	0	0	19.3	189	10.1	99	10.2	100	0.7	7
Romania	44.8	45.0	22.1	874	57.7	2278	20.2	797	41.6	1658	3.8	151	3.6	144	26.5	1057	14.8	592	3.8	151	4.6	184	1.3	53
Russia	42.5	43.0	7.2	362	59.4	3004	33.4	1691	60.3	3046	6.2	311	2.8	139	15.7	793	5.9	300	2.9	144	4.9	249	1.3	66
Rwanda	34.2	32.0	56	1305	33.8	789	10.2	238	17.2	421	7.7	189	50	1226	0.7	18	6.7	165	6.8	168	7.7	188	3.2	79
Serbia	42.9	43.0	19.2	250	58.7	763	22	286	39	505	2.6	34	8	104	16	207	5.4	70	4.9	64	23.2	301	0.8	11
Singapore	40.7	40.0	16.8	280	52.5	873	30.6	509	51	872	9.1	156	5.1	88	8	137	13.5	231	8.2	140	4.3	73	0.8	13
Slovakia	48.3	50.0	13.8	164	74.4	885	11.8	141	51.6	614	2	24	6.1	73	22.3	265	2.4	29	1.5	18	7.4	88	6.6	78
Viet Nam	39.7	38.0	54.5	701	37.2	478	8.3	107	16.6	219	10.9	143	18.8	248	8.3	109	4.9	65	1.5	20	4.3	56	34.7	456
Slovenia	44.0	44.0	27.8	799	46.8	1347	25.4	731	53.4	1541	2	57	4.1	117	21	606	2.8	80	8.1	233	7.3	212	1.5	42
South Africa	38.0	36.0	17.4	950	72.7	3977	9.9	543	34.6	1954	8.4	473	4.8	272	7.6	428	7	393	4.7	265	33	1861	0	1
Zimbabwe	33.9	30.0	24	327	65.1	887	10.9	148	24.7	337	7.9	108	18.4	252	2.6	36	11.6	158	4.6	63	30.2	412	0	0
Spain	42.4	41.0	46.4	1423	34.2	1051	19.4	596	45.9	1424	4.8	150	8.4	262	10.5	327	14.2	439	3.5	107	12.2	378	0.5	14
Sweden	45.7	46.0	11.8	330	42.4	1183	45.8	1276	57.8	1618	11.4	320	4.8	135	7.3	205	4.6	129	6.3	177	3.9	109	3.8	106
Switzerland	46.6	46.0	11.2	231	59	1213	29.7	611	50.6	1041	17.5	361	6	123	12.2	252	8.1	166	1.8	37	1.7	35	2.1	44
Thailand	43.6	44.0	56.8	1383	26.4	642	16.8	408	23.8	591	7	173	54.8	1359	1.3	31	4.8	119	2.2	54	5.4	133	0.8	19
Trinidad and Tobago	42.1	41.0	43.1	733	47.3	805	9.6	164	41.1	705	8.2	140	11	188	10.6	181	12.6	216	1.6	28	14.7	252	0.2	4
Tunisia	38.9	36.0	48.6	402	32.1	266	19.3	160	24.1	254	7.2	76	13.1	138	6.9	73	22.3	235	10.4	110	15.3	161	0.6	6
Turkey	38.3	36.0	55.2	2559	30.3	1405	14.6	676	22.7	1091	4	192	10.2	489	9	432	38.7	1863	5.5	266	9.3	447	0.7	36
Ukraine	43.0	43.0	5	165	54.8	1822	40.2	1337	47.4	1576	8.4	281	4.1	138	20.3	677	7.8	260	2.9	95	7.1	236	2	65
Macedonia	42.1	42.0	16.8	228	58.8	796	24.4	330	38.2	519	2.3	31	3.7	50	11.8	160	4.6	63	8.8	120	29.2	396	1.4	19
Egypt	39.6	38.0	26	796	48.7	1489	25.3	774	29.8	1277	3.3	140	2.4	104	4.1	174	52.6	2253	1.9	82	4.6	195	1.4	59

Country	Age		Education						Employment status															
			Lower		Middle		Upper		Full time		Part time		Self employed		Retired		Housewife		Students		Unemployed		Other	
	Mean	Median	%	n	%	n	%	n	%	n	%	n	%	n	%	n	%	n	%	n	%	n	%	n
Great Britain	44.4	44.0	31.9	620	37.4	728	30.7	596	38.3	776	14.4	291	7.8	158	14.7	298	9.6	195	2.4	49	8.6	174	4.1	83
United States	45.6	47.0	5.7	170	51.2	1526	43.1	1283	50.6	1491	5.9	175	7.1	208	12.9	380	7.8	229	2.2	64	6.8	201	6.8	200
Burkina Faso	35.4	32.0	64.2	442	25.3	174	10.5	72	11	133	4.1	50	25.7	311	3.7	45	26.6	322	5.7	69	18.5	224	4.7	57
Uruguay	42.7	42.0	60.2	973	26.5	429	13.3	215	41.4	672	10.7	174	11	178	12.5	203	12.6	204	1.9	31	9.2	150	0.6	10
Uzbekistan	38.5	37.0	7.3	99	78	1051	14.7	198	22	297	16.1	217	7.8	106	12.1	164	27.1	366	1.4	19	13.1	177	0.4	5
Yemen	35.5	33.0	44.8	245	28.3	155	26.9	147	16.7	153	10.9	100	13.1	120	2.4	22	42.3	387	2.1	19	12.5	114	0	0
Zambia	31.9	28.0	37.6	426	45.7	518	16.8	190	22.6	278	4.6	56	11.4	140	4.4	54	8.8	108	10.7	131	37.1	455	0.5	6

Figure S1. Pro-equality views and full-time employment in 97 countries

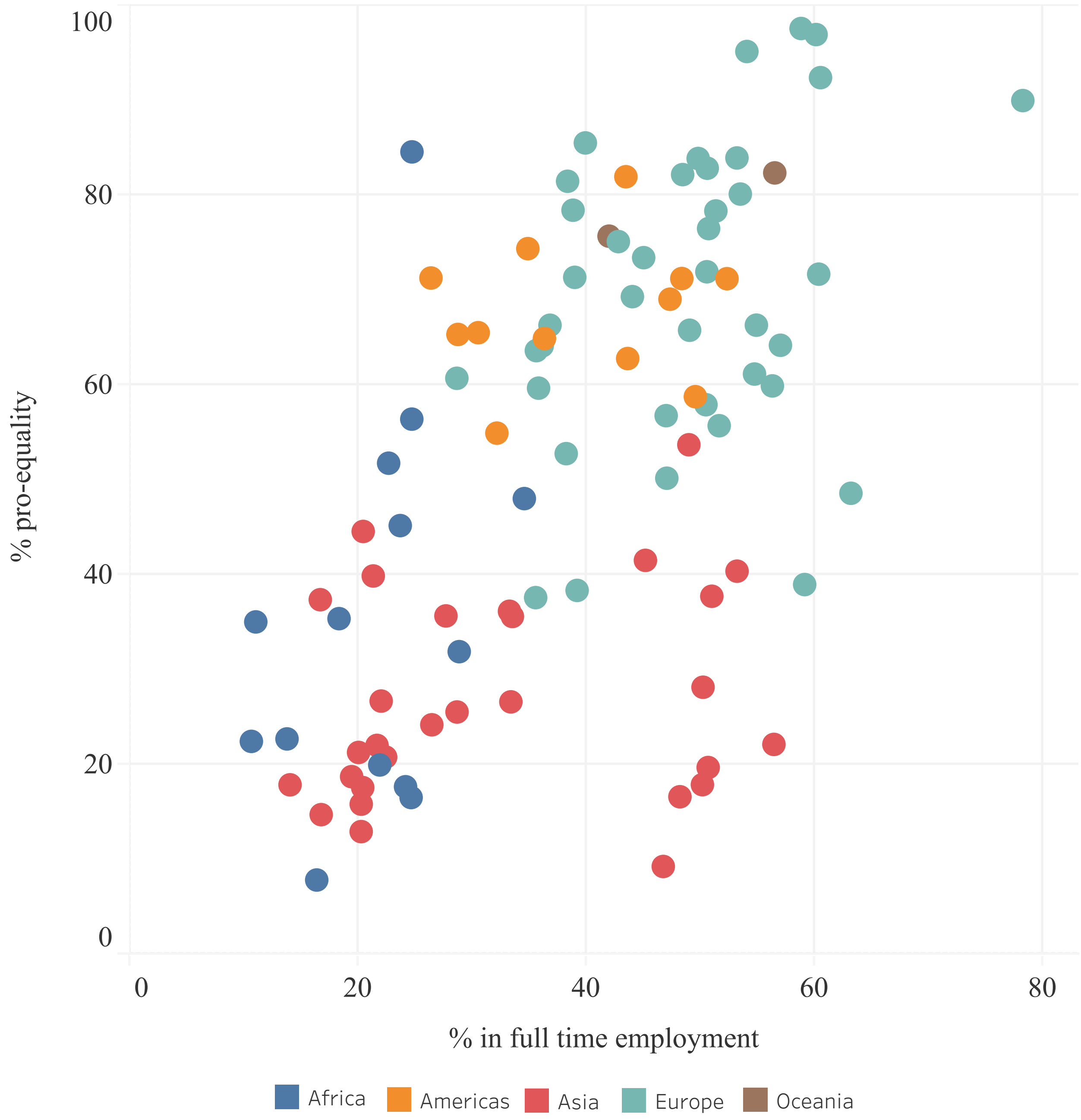

Figure S2: Self-reported health among men and women by full-time employment for quartiles of pro-equality views

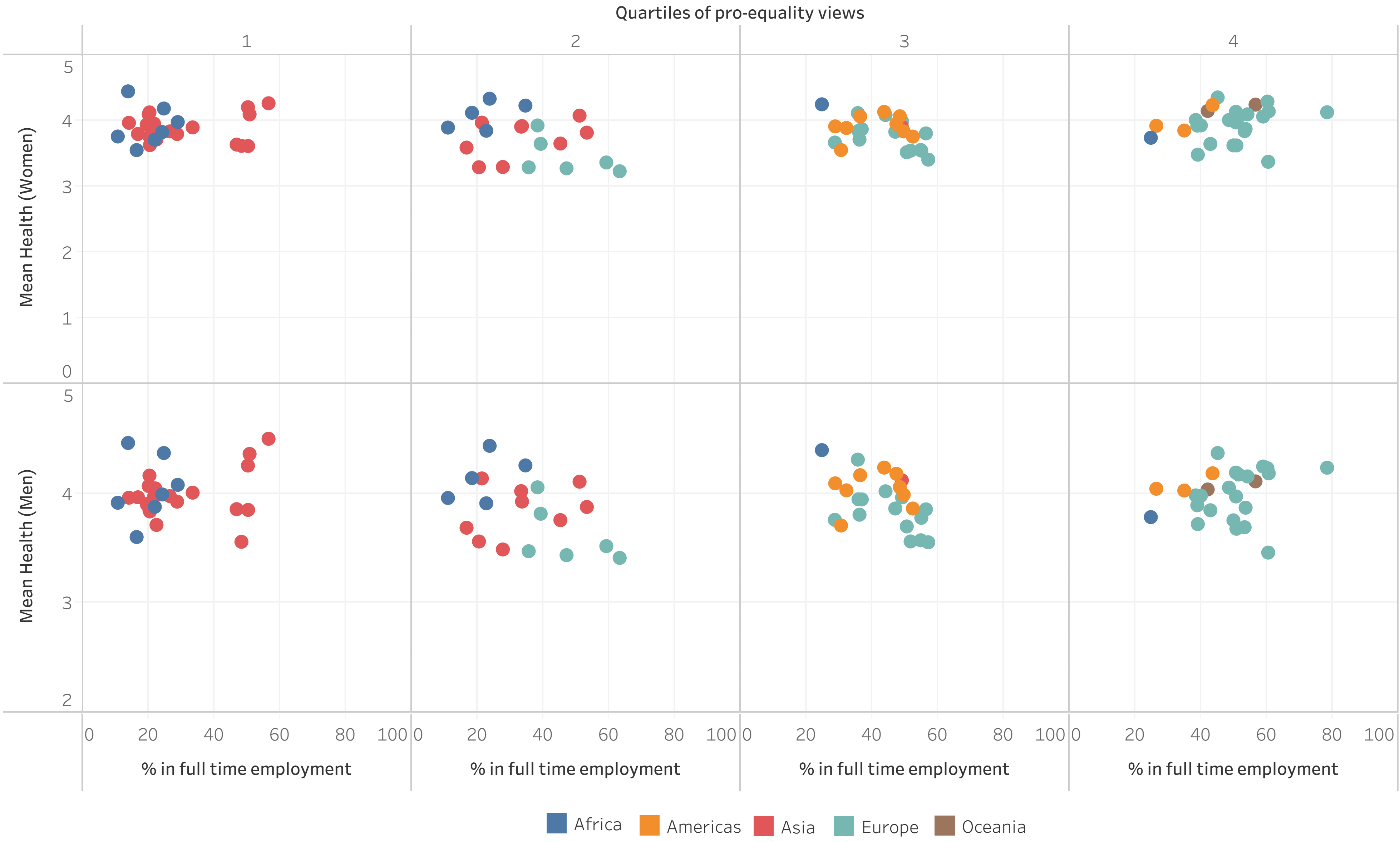

Supplement: Supplementary file 1 [file Data_Sheet_1.pdf]
